# Genome-Wide Assessment of Differential DNA Methylation Associated with Autoantibody Production in Systemic Lupus Erythematosus

**DOI:** 10.1371/journal.pone.0129813

**Published:** 2015-07-20

**Authors:** Sharon A. Chung, Joanne Nititham, Emon Elboudwarej, Hong L. Quach, Kimberly E. Taylor, Lisa F. Barcellos, Lindsey A. Criswell

**Affiliations:** 1 Rosalind Russell / Ephraim P. Engleman Rheumatology Research Center, Division of Rheumatology, University of California San Francisco, San Francisco, California, United States of America; 2 Division of Epidemiology, Genetic Epidemiology and Genomics Laboratory, School of Public Health, University of California, Berkeley, California, United States of America; Instituto Nacional de Ciencias Medicas y Nutricion Salvador Zubiran, MEXICO

## Abstract

Systemic lupus erythematosus (SLE) is characterized by the development of autoantibodies associated with specific clinical manifestations. Previous studies have shown an association between differential DNA methylation and SLE susceptibility, but have not investigated SLE-related autoantibodies. Our goal was to determine whether DNA methylation is associated with production of clinically relevant SLE-related autoantibodies, with an emphasis on the anti-dsDNA autoantibody. In this study, we characterized the methylation status of 467,314 CpG sites in 326 women with SLE. Using a discovery and replication study design, we identified and replicated significant associations between anti-dsDNA autoantibody production and the methylation status of 16 CpG sites (p_discovery_<1.07E-07 and p_replication_<0.0029) in 11 genes. Associations were further investigated using multivariable regression to adjust for estimated leukocyte cell proportions and population substructure. The adjusted mean DNA methylation difference between anti-dsDNA positive and negative cases ranged from 1.2% to 19%, and the adjusted odds ratio for anti-dsDNA autoantibody production comparing the lowest and highest methylation tertiles ranged from 6.8 to 18.2. Differential methylation for these CpG sites was also associated with anti-SSA, anti-Sm, and anti-RNP autoantibody production. Overall, associated CpG sites were hypomethylated in autoantibody positive compared to autoantibody negative cases. Differential methylation of CpG sites within the major histocompatibility region was not strongly associated with autoantibody production. Genes with differentially methylated CpG sites represent multiple biologic pathways, and have not been associated with autoantibody production in genetic association studies. In conclusion, hypomethylation of CpG sites within genes from different pathways is associated with anti-dsDNA, anti-SSA, anti-Sm, and anti-RNP production in SLE, and these associations are not explained by genetic variation. Thus, studies of epigenetic mechanisms such as DNA methylation represent a complementary method to genetic association studies to identify biologic pathways that may contribute to the clinical heterogeneity of autoimmune diseases.

## Introduction

Systemic lupus erythematosus (SLE) is a chronic autoimmune disease that can affect virtually any organ system. The pivotal immunologic disturbance in SLE is the formation of autoantibodies directed against nuclear and cellular components. Autoantibodies recognizing double-stranded DNA (dsDNA) are of particular importance given their clinical relevance in SLE. Anti-dsDNA autoantibodies are observed in 40–60% of SLE patients, implicated in the pathogenesis of lupus nephritis (and thus are more prevalent in patients with lupus nephritis), and associated with decreased survival. Antibodies targeting small nuclear ribonucleoproteins (anti-Sm, anti-RNP) or proteins complexed with small RNAs (anti-SSA/Ro, anti-SSB/La) occur in 10–40% of SLE patients and are associated with musculoskeletal and mucocutaneous manifestations, as well as neonatal heart block [1, 2].

To help determine the pathogenic mechanisms contributing to their production, the genetic basis for autoantibody production in SLE has been examined in both genome-wide and candidate gene association studies. For example, we have previously shown that certain SLE susceptibility loci demonstrate stronger associations with anti-dsDNA autoantibody production than SLE itself [3], and that genetic variation in the major histocompatibility complex (MHC) is more strongly associated with anti-SSA/Ro and anti-SSB/La autoantibody production than other SLE manifestations [4]. However, the genetic variants identified thus far do not fully explain the propensity to produce autoantibodies in SLE. Therefore, in this study, we examined whether variation in epigenetic factors contributes to autoantibody production in SLE.

DNA methylation, an epigenetic modification, can influence gene expression and has been implicated in the pathogenesis of SLE. In DNA methylation, methylation of C-G dinucleotides (CpG) in a gene can lead to decreased or silenced gene expression [5, 6]. T-cells from SLE patients with active disease have decreased DNA methylation compared to T-cells from matched healthy controls [7]. Inhibition of DNA methylation in T-cells can induce a lupus-like disease in mice [8]. Procainamide and hydralazine, both associated with drug-induced lupus, are also known to inhibit DNA methylation [9].

DNA methylation in SLE is just beginning to be studied at a genome-wide level. A study of five monozygotic twin pairs discordant for SLE found differential methylation in genes regulating immune responses, cytokine production, and cell activation [10]. Two recent studies have shown that interferon-regulated genes are hypomethylated in SLE patients compared to unaffected controls [11, 12]. These relatively small studies (the largest with 75 SLE cases) have confirmed the hypothesis that differential DNA methylation is, indeed, associated with SLE susceptibility. However, these studies have not examined whether DNA methylation is associated with specific disease manifestations. Therefore, we conducted this study, one of the largest studies of DNA methylation in SLE to date, to determine whether DNA methylation is associated with autoantibody production in SLE, with a specific emphasis on the clinically important anti-dsDNA autoantibody. Our results indicate that differential DNA methylation of multiple genomic regions is associated with clinically relevant autoantibody production in SLE.

## Materials and Methods

### Ethics statement

Written informed consent was obtained from all study participants and the institutional review board at the University of California, San Francisco, approved the study.

### Study subjects

All SLE cases studied (n = 326) were participants of the University of California, San Francisco Lupus Genetics Project [13] and fulfilled at least four American College of Rheumatology classification criteria for SLE [14, 15] as determined by medical record review. All participants were women of European descent based on four grandparental countries of origin, and had never smoked based on questionnaire responses.

Anti-dsDNA, anti-Sm, anti-RNP, anti-SSA/Ro and anti-SSB/La autoantibody status was determined by medical record review and/or testing of banked serum. An SLE case had to have at least one definitively positive laboratory result for a particular autoantibody to be considered positive for that autoantibody. An SLE subject was considered negative for a specific autoantibody if all laboratory results in the medical record and serum testing for that autoantibody were negative.

### DNA methylation profiling

Genomic DNA was isolated from the peripheral blood of each participant as previously described [13]. For each sample, 750 ng of DNA underwent bisulfite conversion using the Zymo EZ-96DNA Methylation Kit (Catalog #D5004). DNA methylation profiling was performed using the Infinium HumanMethylation450 BeadChip (Illumina, Inc., San Diego, CA), which was processed according to the manufacturer’s protocol. This high-throughput array profiles >485,000 CpG sites in ~23,000 genes and assesses methylation sites in promoters, 5’ and 3’ regions, gene bodies, CpG islands, CpG island shores, and outside of CpG islands. For this assay, amplified bisulfite converted DNA was interrogated at each locus by fluorescent labeling of two probes: one for sequences of unmethylated DNA, and the other for methylated DNA. The DNA methylation level (reported as “beta”) was calculated as a ratio of methylated signal to total signal intensity. Beta ranges from 0 to 1 (0 = completely unmethylated; 1 = completely methylated).

Beta values were normalized using two methods: primary analyses utilized data that were background subtracted and normalized to internal controls based on the Illumina GenomeStudio software. Analysis was also performed using data that underwent “peak-control” normalization [16] performed in the R package “IMA” [17] to ensure that significant findings were not confounded by differences between the Infinium assay design types.

Several quality control measures were employed. Proper bisulfite conversion, staining, and specificity of internal controls were assessed by the GenomeStudio software. Beta values with a detection p>0.01 were set to missing, where the detection p-value represents the chance that the methylation signal was not distinguishable from negative controls. CpG sites with >5% missing data after quality control filtering (n = 1,164), on the Y-chromosome, or with a single nucleotide polymorphism (SNP) with a minor allele frequency >0.05 located within 50 bp up or downstream [18] (n = 16,953) were removed from analysis. All samples had <5% missing methylation values. Assay reproducibility was validated by comparing duplicate samples. For each of the 38 duplicate pairs, the Pearson correlation of beta values across all CpG sites was greater than 0.997. After quality control filtering, 326 samples and 467,314 CpG sites were analyzed.

### Cell Population Estimation

Since the DNA was obtained from peripheral blood leukocytes and DNA methylation can differ between leukocyte cell types [19], we estimated the leukocyte cell proportions in the samples using regression calibration as presented by Houseman *et al*. [20] and used in an epigenetic study of rheumatoid arthritis [21]. This algorithm predicts the proportion of specific cell populations in whole blood based on the beta values for CpG sites previously found to be informative for specific leukocyte subsets. We also used the validation dataset accompanying the Houseman *et al*. study, which was generated on the Infinium HumanMethylation27 BeadChip. Of the 500 most informative CpG sites in the validation dataset, 472 sites were on the HumanMethylation450 BeadChip. The beta values for these CpG sites were used to estimate the proportion of granulocytes, monocytes, B cells, T cells and NK cells for each sample.

### Population substructure

Since DNA methylation differences have been observed between ethnic groups [22], we adjusted for population substructure in multivariable analyses. All subjects were previously genotyped on the Illumina HumanHap500 BeadChip [23] (n = 264), Illumina ImmunoChip (n = 289), or both (n = 227). Between the two panels, 24,873 SNPs were genotyped in common and met standard quality control measures. This set of SNPs was LD-pruned (r^2^<0.2) using PLINK [24] to 12,816 SNPs. Genotype data for these SNPs were merged using PLINK. Concordance rates for the SNP genotypes from the individuals characterized on both panels was >99.96%. Principal components analysis was implemented in EIGENSTRAT [25] using the 12,816 SNPs. The first principal component was included in multivariable analyses to adjust for population substructure (see below). No individuals were identified as genetic outliers.

### Statistical Analysis

For the initial analyses focused on anti-dsDNA autoantibody production, SLE cases were separated into discovery (n = 186) and replication (n = 140) cohorts, based on whether the sample was in the 1^st^ or 2^nd^ batch of assays performed. Each batch had both anti-dsDNA autoantibody positive and negative cases: the discovery cohort (batch 1) had 94 anti-dsDNA positive and 92 anti-dsDNA negative SLE cases, while the replication cohort (batch 2) had 62 anti-dsDNA positive and 78 anti-dsDNA negative SLE cases. Wilcoxon rank sum tests were used to identify CpG sites with statistically significant differences in methylation between anti-dsDNA positive and anti-dsDNA negative SLE cases in both the discovery and replication cohorts. The threshold for statistical significance for the discovery cohort was set at p<1.07E-07, based on the Bonferroni correction (0.05/467,314 = 1.07E-07), which is more stringent than the false-discovery rate method. The 17 CpG sites that achieved statistical significance in the discovery dataset were further assessed in the replication dataset, with p<0.05/17 = 0.0029 considered statistically significant. The discovery and replication datasets were combined (n = 326) in multivariable analyses. For logistic regression analyses, beta values for each CpG site were divided into tertiles. The highest and lowest tertiles were compared for association with anti-dsDNA status adjusting for age at the time of sample, sample plate, disease duration, population substructure, and estimated leukocyte cell proportions. Linear regression models adjusting for the same covariates were used to estimate the adjusted mean differences in DNA methylation for each site between the anti-dsDNA positive and negative groups. These analyses were performed in R version 3.0.1 [26]. The same linear regression modeling technique was used to assess differences in DNA methylation associated with anti-Sm, anti-RNP, and anti-SSA/Ro autoantibody production. Anti-SSB/La was not analyzed since 30 out of 33 anti-SSB/La positive individuals were also anti-SSA/Ro positive.

To examine the relationship between DNA methylation and genetic variation for anti-dsDNA autoantibody production, we utilized SNP data available from a GWAS of anti-dsDNA autoantibody production published by our group [3]. We identified SNPs on the Illumina HumanHap 500 Beadchip located 250 kb up- and downstream of the associated CpG sites. We assessed each SNP’s association with anti-dsDNA autoantibody production by comparing 811 anti-dsDNA positive to 906 anti-dsDNA negative SLE cases (all of European descent). Details of the statistical analysis and quality control measures are presented in Chung *et al*. [3]. For SNPs associated with anti-dsDNA autoantibody production, we examined the correlation between DNA methylation status and SNP genotype coded as an additive model. Evidence of interaction between SNPs and methylation status in the 264 SLE cases genotyped on the Illumina HumanHap500 Beadchip was assessed using logistic regression models implemented in STATA (College Station, TX). These models included SNP genotype, methylation tertile, a genotype/methylation interaction term, and the previously described covariates.

## Results

### Assessment of differential DNA methylation

**Fig 1:**
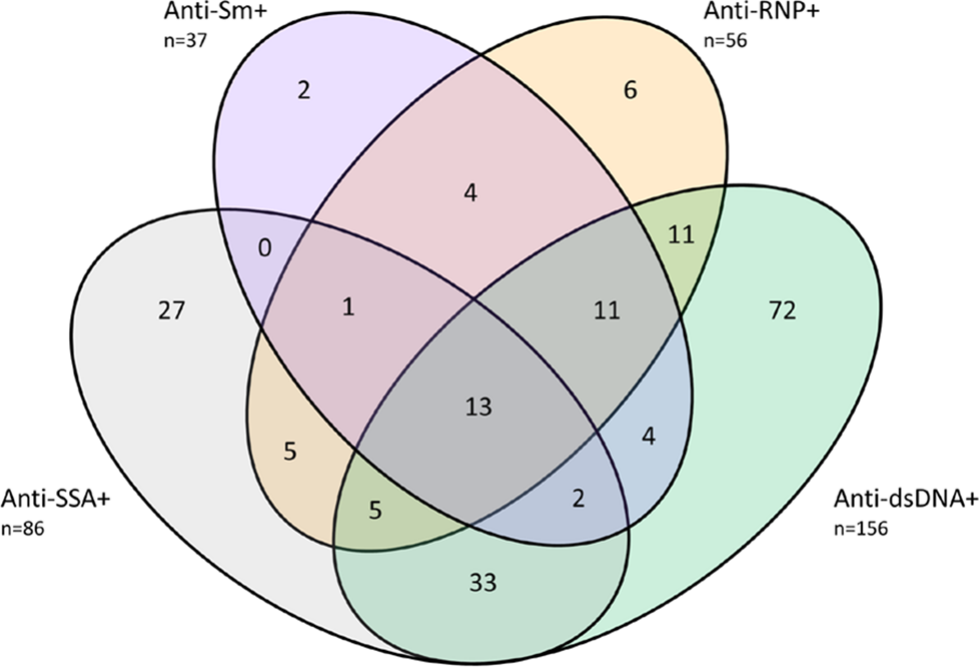
Autoantibody distribution of the study participants (n = 326).

The clinical characteristics of the 326 SLE cases examined in this study are presented in Table 1 and Fig 1. To minimize confounding, we studied only women of European descent who had never smoked. All 326 SLE cases were successfully characterized on the HumanMethylation450 BeadChip and data for 467,314 CpG sites were analyzed.

**Table 1:** Clinical characteristics of the 326 SLE cases in the study.

Characteristic	
Age at DNA sample, mean years (SD)	46 (13)
Disease duration, median years (IQR)	10 (6–17)
ACR classification criteria for SLE, mean n (SD)	5 (1)
ACR classification criteria, n (%):	
Malar rash	153 (47)
Discoid rash	10 (3)
Photosensitivity	263 (81)
Oral ulcers	99 (30)
Arthritis	247 (76)
Serositis	96 (29)
Neurologic	30 (9)
Immunologic	218 (67)
Hematologic	219 (67)
Renal	80 (25)
Anti-nuclear antibody (ANA)	309 (95)
SLE-related autoantibodies, n (%):	
Anti-dsDNA	156 (48)
Anti-SSA/Ro	86 (27^a^)
Anti-SSB/La	33 (10^a^)
Anti-Sm	37 (12^a^)
Anti-RNP	56 (17^a^)

SD = standard deviation

IQR = interquartile range

ACR = American College of Rheumatology

^a^ percentage may not be based on 326 individuals due to individuals with unknown status

Our primary analyses focused on the anti-dsDNA autoantibody given its clinical significance, and our previous work indicating that anti-dsDNA positive and negative cases are genetically different [3]. To identify CpG sites most robustly associated with anti-dsDNA status, we employed a discovery and replication study design. Using Wilcoxon rank sum tests, 17 sites showed statistical evidence of association (p<1.07E-07) with anti-dsDNA autoantibody status in the discovery dataset (Table 2). Replication of this association was achieved for 16 sites (p<0.0029). Across all 16 CpG sites, anti-dsDNA positive SLE cases were less methylated than anti-dsDNA negative SLE cases and the difference in methylation ranged from 1.5%-24% in the combined dataset. Essentially the same CpG sites were identified using the peak-control normalized data (data not shown), and thus the original background-subtracted/control-normalized data were used for all analyses. These 16 sites were located in 11 genomic regions (Table 2). Of note, none of these 16 sites were located in the major histocompatibility complex.

**Table 2:** DNA methylation sites significantly associated with anti-dsDNA autoantibody production using the discovery/replication study design.

				Discovery (n = 186)		Replication (n = 140)		Combined (n = 326)		Linear Regression (n = 326)		Logistic Regression (n = 326)	
site	chr	position	Gene(s)	p	Mean difference^a^	p	Mean difference^a^	p	Mean difference^a^	p	Mean difference^b^ (95% CI)	p	OR (95% CI)^c^
**cg05552874**	10	91153143	*IFIT1*	1.4E-09	-0.15	1.7E-10	-0.17	6.5E-18	-0.16	7.8E-13	-0.11 (-0.14 to -0.084)	8.4E-09	15.0 (6.0–37.9)
**cg06872964**	1	79085250	*IFI44L*	5.7E-10	-0.13	4.1E-10	-0.147	7.2E-18	-0.14	2.0E-13	-0.10 (-0.13 to -0.077)	3.9E-09	13.7 (5.7–32.8)
**cg21549285**	21	42799141	*MX1*	1.1E-09	-0.23	1.3E-09	-0.25	3.0E-17	-0.24	1.4E-13	-0.19 (-0.24 to -0.14)	1.1E-10	16.6 (7.08–39.0)
**cg00959259**	3	122281975	*PARP9*	3.3E-10	-0.16	1.6E-08	-0.18	1.8E-16	-0.16	2.3E-11	-0.13 (-0.16 to -0.091)	8.8E-09	12.6 (5.3–30.0)
**cg10959651**	2	7018020	*RSAD2*	2.1E-10	-0.066	3.4E-07	-0.063	4.4E-16	-0.065	3.2E-13	-0.052 (-0.065 to -0.039)	1.4E-09	13.6 (5.9–31.8)
**cg13130398**	1	174844397	*RABGAP1L*	6.3E-09	-0.088	5.1E-08	-0.093	4.4E-15	-0.089	5.2E-11	-0.073 (-0.094 to -0.052)	2.5E-09	10.5 (4.8–22.6)
**cg07285983**	1	174844490	*RABGAP1L*	4.2E-09	-0.11	1.4E-07	-0.11	6.9E-15	-0.11	3.5E-12	-0.088 (-0.11 to -0.064)	5.3E-08	7.9 (3.8–16.6)
**cg05696877**	1	79088769	*IFI44L*	1.2E-08	-0.18	2.7E-08	-0.21	7.7E-15	-0.19	1.3E-13	-0.16 (-0.19 to -0.12)	1.5E-10	18.2 (7.5–44.2)
**cg07839457**	16	57023022	*NLRC5*	1.6E-08	-0.14	9.4E-08	-0.15	1.2E-14	-0.14	4.6E-13	-0.12 (-0.15 to -0.087)	3.3E-09	13.6 (5.7–32.3)
**cg08122652**	3	122281939	*PARP9; DTX3L*	8.7E-08	-0.14	3.9E-08	-0.15	3.7E-14	-0.15	2.4E-09	-0.11 (-0.14 to -0.077)	4.2E-09	12.7(5.4–29.6)
**cg10549986**	2	7018153	*RSAD2*	8.3E-09	-0.058	9.0E-07	-0.055	4.3E-14	-0.056	3.3E-11	-0.044 (-0.056 to -0.031)	6.7E-06	8.2 (3.7–17.9)
**cg06981309**	3	146260954	*PLSCR1*	4.7E-08	-0.11	1.6E-07	-0.11	4.3E-14	-0.11	1.0E-10	-0.074 (-0.096 to -0.053)	1.5E-07	6.8 (3.0–15.6)
**cg01948202**	3	122400474	*PARP14*	2.2E-08	-0.059	3.5E-07	-0.053	4.8E-14	-0.056	1.7E-07	-0.042 (-0.058 to -0.027)	2.7E-07	8.3 (3.71–18.7)
**cg17326313**	2	37383568	*EIF2AK2*	9.4E-08	-0.033	1.1E-06	-0.038	6.5E-13	-0.035	3.5E-09	-0.022 (-0.029 to -0.015)	2.8E-06	7.8 (3.3–18.5)
**cg19789466**	12	113344923	*OAS1*	2.3E-08	-0.016	4.6E-06	-0.015	6.7E-13	-0.015	7.2E-10	-0.012 (-0.016 to -0.0083)	9.6E-08	7.9 (3.7–16.9)
**cg16411857**	16	57023191	*NLRC5*	3.3E-08	-0.055	6.2E-06	-0.051	1.7E-12	-0.053	2.0E-10	-0.045 (-0.058 to -0.032)	5.1E-08	11.0 (4.6–26.0)
**cg12667031**	1	169878852	-	8.0E-08	0.0065	0.015	0.0031	did not replicate					

^a^ Unadjusted difference in DNA methylation between anti-dsDNA positive and anti-dsDNA negative SLE cases [methylation(anti-dsDNA positive)-methylation(anti-dsDNA negative)]

^b^ Mean difference represents the coefficient from the linear regression model which indicates the difference in DNA methylation between anti-dsDNA positive and negative SLE cases adjusted for age, disease duration, sample plate, population substructure, and estimated leukocyte proportions

^c^ OR represents the odds of being anti-dsDNA autoantibody positive if an individual’s DNA methylation level is in the lowest tertile compared to the highest tertile, adjusted for age, disease duration, sample plate, population substructure, and estimated leukocyte proportions

For the combined group, 183 CpG sites showed significant evidence of association (p<1.07E-07) (S1 Table). A total of 11,557 CpG sites were associated with anti-dsDNA autoantibody production at p<0.001, when 467 sites would be expected by chance alone. This result indicates a substantial enrichment of associated CpG sites in our data and demonstrates that DNA methylation is associated with anti-dsDNA autoantibody production. S1 Fig presents a volcano plot indicating the methylation difference and associated p-value for each CpG site.

**Fig 2:**
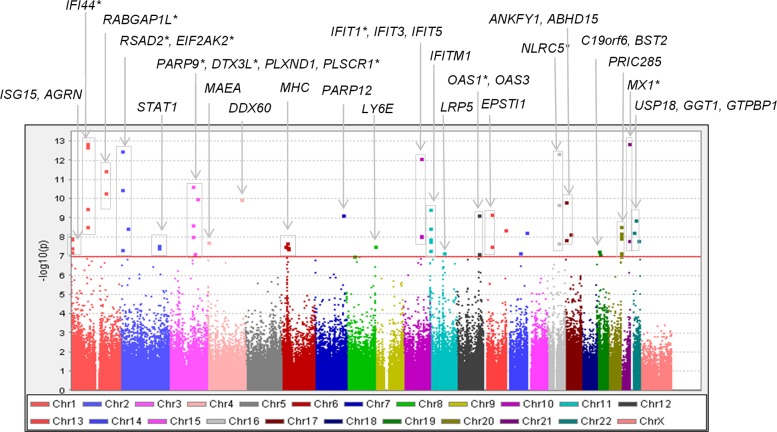
Manhattan plot of the DNA methylation sites associated with anti-dsDNA autoantibody production. Results are based on multivariable linear regression for the combined dataset. The red line indicates a significance level of p<1.07E-07. Points above the red line that are unannotated are not within a known gene. Genes marked with an asterisk (*) were associated with anti-dsDNA autoantibody production using the discovery/replication study design.

Since DNA methylation can be influenced by age [27], tissue/cell type [19], and ethnicity [22], we employed multivariable regression to adjust for these and other potential confounders. Leukocyte proportions were estimated using regression calibration as presented by Houseman *et al*. [20]. The multivariable linear regression results shown in Table 2 present the adjusted mean difference between anti-dsDNA positive and negative SLE cases for the 16 sites identified using the discovery/replication study design (see Materials and Methods). A Manhattan plot showing the adjusted associations for the combined dataset is presented in Fig 2. The logistic regression results are quite striking—when comparing the lowest tertile to the highest tertile of methylation, the odds of anti-dsDNA autoantibody production are 6.8–18.2 times higher for the lowest tertile (Table 2).

### Genetic associations and genetic-epigenetic interactions

Since genetic variation can be correlated with DNA methylation [28], we examined whether the associations observed with the 11 genomic regions presented in Table 2 could be explained by genetic variation. Using genotype information available from a genome-wide association study of anti-dsDNA autoantibody production [3], we identified 898 SNPs that were located within 250 kb up- and downstream of the differentially methylated sites presented in Table 2. Only 35 of the 898 SNPs identified showed marginal evidence of association (p<0.05), and only 2 SNPs (located in or near *OAS1* and *NLRC5*) were associated with anti-dsDNA autoantibody production at a p<0.01 threshold (S2 Table). These two SNPs were not correlated with their surrounding associated CpG sites (Pearson r <0.1 all SNP/methylation site pairs). Logistic regression was used to assess for evidence of an interaction between the methylation status of sites near these two SNPs and the SNP genotype (n = 264, see Materials and Methods). No evidence of interaction (p>0.05) was observed for either gene (data not shown).

### DNA methylation differences observed with other SLE-related autoantibodies

We next examined whether these results were relevant for other SLE-related autoantibodies, or were specific for anti-dsDNA autoantibody production. We focused our analyses on anti-SSA/Ro, anti-Sm, and anti-RNP (see Materials and Methods), and the distribution of autoantibody positivity among the 326 SLE cases is presented in Fig 1. Out of the 326 SLE cases, 121 (37%) individuals were negative for all four autoantibodies. The status of at least one autoantibody was unknown for 9 individuals.

Using multivariable linear regression performed in the same manner as for the anti-dsDNA autoantibody analyses, we examined the difference in methylation for the 16 CpG sites presented in Table 2 across the other SLE-related autoantibodies using the combined dataset. Table 3 shows that methylation was significantly different (p<1.07E-07) between those positive and negative for the other SLE-related autoantibodies for all but two sites: cg19789466 (*OAS1*) and cg17326313 (*EIF2AK2*). Methylation differences for these two sites were statistically significant for three of the SLE-related autoantibodies; the methylation difference was of a similar magnitude but not statistically significant for the fourth autoantibody. For all 16 CpG sites, the observed difference in methylation for the anti-Sm and anti-RNP analyses was larger than the difference seen in the anti-dsDNA and anti-SSA/Ro analyses. These findings indicate that differential methylation of these genes is associated with SLE-related autoantibody production in general, and not linked to a specific autoantibody.

**Table 3:** DNA methylation differences for anti-SSA/Ro, anti-Sm, and anti-RNP autoantibody production for sites significantly associated with anti-dsDNA autoantibody production.

		Anti-dsDNA		Anti-SSA/Ro		Anti-Sm		Anti-RNP	
site	Gene	Mean difference^a^ (95% CI)	p	Mean difference^a^ (95% CI)	p	Mean difference^a^ (95% CI)	p	Mean difference^a^ (95% CI)	p
**cg05552874**	*IFIT1*	-0.11	7.8E-13	-0.10	2.6E-08	-0.18	1.4E-11	-0.17	4.6E-16
		(-0.14 to -0.084)		(-0.14 to -0.067)		(-0.23 to -0.13)		(-0.21 to -0.13)	
**cg06872964**	*IFI44L*	-0.10	2.0E-13	-0.096	4.8E-09	-0.15	6.4E-10	-0.15	8.5E-15
		(-0.13 to -0.077)		(-0.13 to -0.065)		(-0.19 to -0.10)		(-0.18 to -0.11)	
**cg21549285**	*MX1*	-0.19	1.4E-13	-0.18	8.0E-09	-0.27	5.5E-10	-0.27	2.0E-14
		(-0.24 to -0.14)		(-0.23 to -0.12)		(-0.36 to -0.19)		(-0.33 to -0.20)	
**cg00959259**	*PARP9*	-0.13	2.3E-11	-0.14	1.3E-10	-0.18	2.6E-08	-0.18	8.2E-13
		(-0.16 to -0.091)		(-0.18 to -0.099)		(-0.24 to -0.12)		(-0.23 to -0.13)	
**cg10959651**	*RSAD2*	-0.052	3.2E-13	-0.052	2.9E-10	-0.071	3.6E-09	-0.071	9.3E-14
		(-0.065 to -0.039)		(-0.067 to -0.036)		(-0.094 to -0.048)		(-0.089 to -0.053)	
**cg13130398**	*RABGAP1L*	-0.073	5.2E-11	-0.064	7.1E-07	-0.10	4.5E-08	-0.096	1.3E-10
		(-0.094 to -0.052)		(-0.088 to -0.039)		(-0.14 to -0.066)		(-0.12 to -0.068)	
**cg07285983**	*RABGAP1L*	-0.088	3.5E-12	-0.081	3.3E-08	-0.13	3.0E-10	-0.12	2.2E-13
		(-0.11 to -0.064)		(-0.11 to -0.053)		(-0.17 to -0.093)		(-0.16 to -0.092)	
**cg05696877**	*IFI44L*	-0.16	1.3E-13	-0.15	3.4E-09	-0.17	2.6E-06	-0.19	3.6E-11
		(-0.20 to -0.12)		(-0.19 to -0.099)		(-0.24 to -0.10)		(-0.24 to -0.14)	
**cg07839457**	*NLRC5*	-0.12	4.6E-13	-0.12	7.9E-10	-0.14	5.3E-07	-0.14	5.9E-10
		(-0.15 to -0.087)		(-0.15 to -0.08)		(-0.19 to -0.085)		(-0.18 to -0.095)	
**cg08122652**	*PARP9;*	-0.11	2.4E-09	-0.12	2.7E-08	-0.22	1.3E-12	-0.20	1.4E-16
	*DTX3L*	(-0.15 to -0.077)		(-0.16 to -0.079)		(-0.28 to -0.16)		(-0.25 to -0.16)	
**cg10549986**	*RSAD2*	-0.044	3.3E-11	-0.047	4.5E-10	-0.051	4.8E-06	-0.060	1.5E-11
		(-0.056 to -0.031)		(-0.062 to -0.033)		(-0.073 to -0.03)		(-0.077 to -0.043)	
**cg06981309**	*PLSCR1*	-0.074	1.0E-10	-0.079	2.9E-09	-0.12	3.8E-09	-0.12	8.3E-14
		(-0.096 to -0.053)		(-0.10 to -0.053)		(-0.15 to -0.078)		(-0.14 to -0.086)	
**cg01948202**	*PARP14*	-0.042	1.7E-07	-0.032	7.5E-04	-0.057	2.7E-05	-0.047	2.3E-05
		(-0.058 to -0.027)		(-0.05 to -0.013)		(-0.084 to -0.031)		(-0.068 to -0.025)	
**cg17326313**	*EIF2AK2*	-0.022	3.5E-09	-0.024	2.4E-08	-0.026	2.8E-05	-0.028	4.5E-08
		(-0.029 to -0.015)		(-0.032 to -0.016)		(-0.039 to -0.014)		(-0.038 to -0.018)	
**cg19789466**	*OAS1*	-0.012	7.2E-10	-0.012	6.3E-08	-0.017	3.4E-07	-0.017	1.1E-10
		(-0.016 to -0.0083)		(-0.016 to -0.0078)		(-0.023 to -0.010)		(-0.022 to -0.012)	
**cg16411857**	*NLRC5*	-0.045	2.0E-10	-0.045	4.0E-08	-0.049	4.3E-05	-0.053	4.2E-08
		(-0.058 to -0.032)		(-0.061 to -0.029)		(-0.073 to -0.026)		(-0.071 to -0.034)	

^a^ The mean difference represents the difference in DNA methylation between autoantibody positive and autoantibody negative SLE cases [methylation(anti-dsDNA positive)-methylation(anti-dsDNA negative)] as assessed by multivariable linear regression.

**Fig 3:**
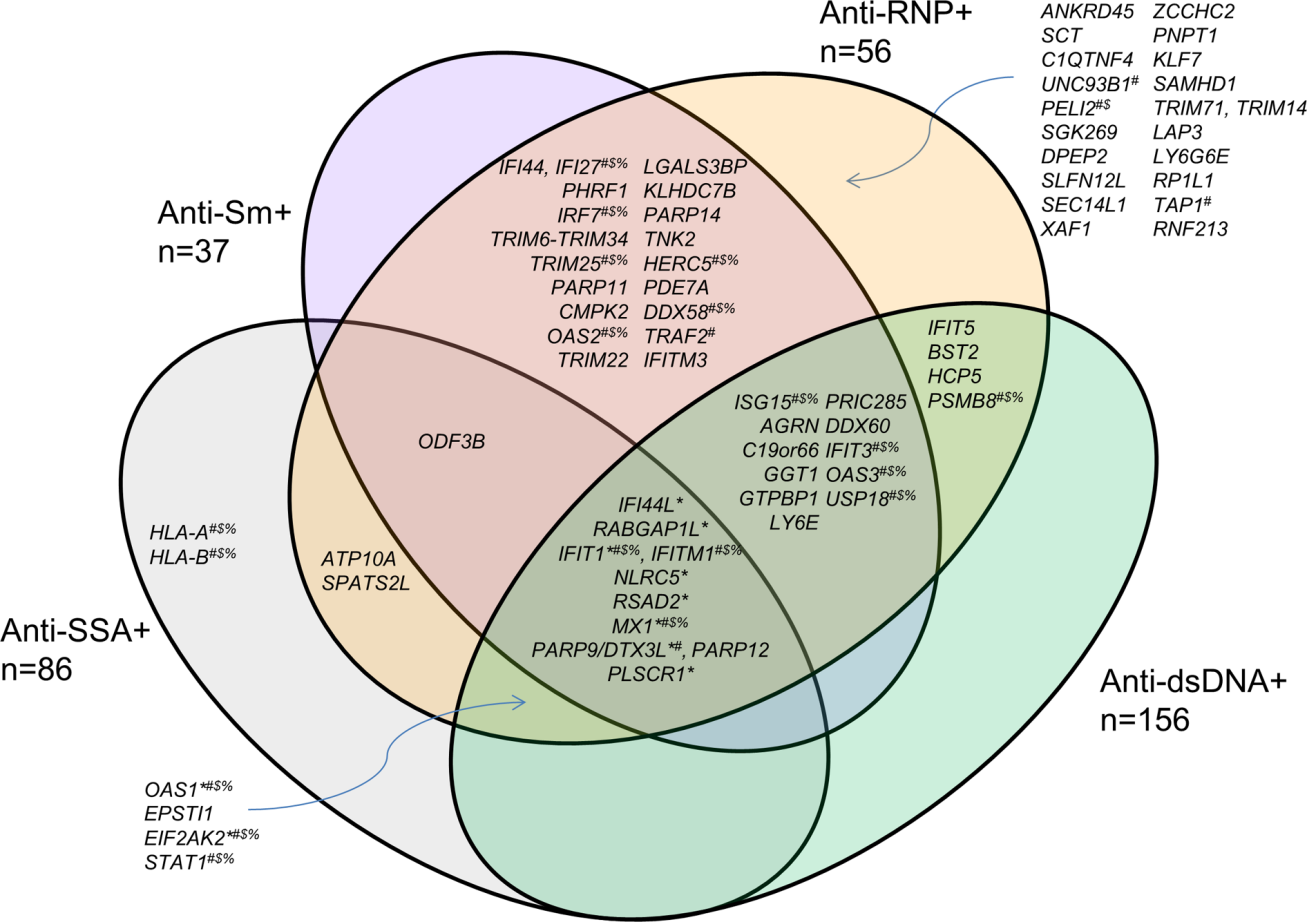
Overlap of genes whose DNA methylation status is significantly associated with at least one SLE-related autoantibody. Results are based on multivariable linear regression analyses using the combined dataset. * Identified in anti-dsDNA discovery/replication analyses_; #_ REACTOME immune system gene; _$_ REACTOME cytokine signaling gene; _%_ REACTOME interferon signaling gene.

Given the overlap in associations observed for these 16 CpG sites, we then examined whether differential methylation of sites within other genes across the genome was associated with the production of multiple SLE-related autoantibodies. Using multivariable linear regression modeling to examine each CpG site characterized on the HumanMethylation450 array, we found that methylation of 28 sites was significantly associated with anti-SSA/Ro autoantibody production, 106 sites were associated with anti-Sm autoantibody production, 162 sites were associated with anti-RNP autoantibody production, and 67 sites were associated with anti-dsDNA autoantibody production (all p<1.07E-07). Out of the 197 CpG sites associated with at least one autoantibody, 112 sites were associated with at least two autoantibodies, indicating substantial overlap between the association results (S3 Table). These 197 CpG sites were located in 87 genes/genomic regions, and Fig 3 shows the genomic regions associated with each autoantibody.

Using the GSEA Molecular Signatures Database (http://www.broadinstitute.org/gsea/msigdb/annotate.jsp), these 87 genes were compared to gene sets from the BIOCARTA (http://www.biocarta.com/genes/index.asp), KEGG (http://www.genome.jp/kegg/pathway.html), and REACTOME (http://www.reactome.org/) pathway databases. The three most-represented pathways among these genes are those involved in the immune system (25 genes, p = 0, FDR q = 0), cytokine signaling (21 genes, p = 0, FDR q = 0), and interferon signaling (20 genes, p = 0, FDR q = 0), and all were from the REACTOME database.

The difference in methylation for a particular CpG site was highly correlated between the four autoantibodies (Pearson correlation coefficient r = 0.94–0.99), and even more tightly correlated than autoantibody status among the SLE cases studied (r = 0.13–0.58). Methylation differences observed in the anti-Sm and anti-RNP analyses were the most tightly correlated (r = 0.99, p<0.00005), followed by differences observed with anti-dsDNA and anti-SSA/Ro autoantibodies (r = 0.98, p<0.0005).

## Discussion

This study is the first DNA methylation study to focus on autoantibody manifestations of SLE. Our findings indicate that the methylation status of CpG sites within 11 genomic regions is robustly associated with anti-dsDNA autoantibody production, and that these methylation differences are also observed in anti-SSA/Ro, anti-Sm, and anti-RNP production.

One major finding of our study is that the genomic regions we identified have not been previously associated with SLE-related autoantibody production in genome-wide or candidate gene association studies. Thus, the associations between DNA methylation status and anti-dsDNA autoantibody production are not readily explained by genetic variation within these genes, as assessed by SNP genotyping platforms. In fact, only one gene in the 11 genomic regions has been associated with SLE susceptibility in a GWAS—*RABGAP1L*. Deletion variants of *RABGAP1L* have been associated with SLE susceptibility in a Korean population [29]. These findings suggest that DNA methylation and other epigenetic studies may identify genes and biologic pathways associated with disease that are not implicated by GWAS.

Another finding of note is the paucity of strong associations between differential methylation of CpG sites within major histocompatibility complex (MHC) genes and autoantibody production in SLE. The MHC region contains the strongest genetic signals for SLE susceptibility [23, 30], and is also strongly associated with SLE-related autoantibody production [3, 4]. Differential methylation of MHC genes is also associated with rheumatoid arthritis susceptibility [21]. However, our results suggest that differential methylation within the MHC region is not strongly associated with autoantibody production among SLE patients. In linear regression analyses, only four CpG sites in two genes—*HCP5* in MHC Class III and *PSMB8* in MHC class II—show statistically significant evidence of association with anti-dsDNA autoantibody production. Differential methylation of these two genes, along with *LY6G6E* in MHC Class III and *TAP1* in MHC Class II is associated with anti-RNP production. Associations with methylation of CpG sites within or near the classical HLA genes are only observed for anti-SSA/Ro autoantibody production—specifically *HLA-A* and *HLA-B*. No MHC region DNA methylation associations were observed for anti-Sm. Of note, each of the classical HLA genes are represented by more CpG sites (26–66 sites) than the average number of sites per gene (17 sites/gene) across the genome, and the density of coverage is higher in the MHC than other genomic regions (S2 Fig). Thus, these findings are likely not due to a lack of coverage for the MHC on the Infinium HumanMethylation450 BeadChip.

In contrast, a large number of strong associations with differential methylation of CpG sites within non-MHC genes were observed for all autoantibodies studied (as seen in Fig 2). When examining across the four SLE-related autoantibodies studied, 87 genomic regions were associated with at least one autoantibody. It is interesting to note that only nine genomic regions were not associated with either anti-Sm or anti-RNP. In addition, both anti-Sm and anti-RNP had more statistically significant associations than anti-dsDNA or anti-SSA even though the number of SLE cases with these autoantibodies was substantially smaller. For the eleven genomic regions identified using the discovery/replication study design, the difference in DNA methylation was larger (and the statistical evidence of association stronger) for the anti-Sm and anti-RNP autoantibody case subgroup comparisons. These results suggest that DNA methylation status may be most correlated with the production of these autoantibodies, and that DNA methylation may have a stronger influence on the propensity to produce anti-Sm and anti-RNP autoantibodies.

In the discovery/replication analysis, we observed associations with CpG sites within genes that are either induced by type 1 interferon (*IFIT1*, *IFI44L*, *MX1*, *RSAD2*, *OAS1*, *EIF2AK2*) or regulate type 1 interferon signaling (*NLRC5*). When examining the 87 genes/genomic regions that were associated with at least one autoantibody, interferon signaling was also one of the most represented pathways. The prominence of DNA methylation changes in interferon-related genes is not unexpected, given the increased expression of interferon-related genes seen in SLE, often called the “interferon signature” [31, 32]. Among SLE patients, increased interferon expression is associated with SLE-related autoantibody production [33–35]. This result provides a proof-of-principle—that the DNA methylation changes observed in the current study reflect the gene expression differences previously observed in specific manifestations of SLE.

New associations between autoantibody production and biologic pathways not related to interferon signaling were also identified. For example, hypomethylation within the *PARP9/DTX3L* region was associated with all 4 autoantibodies studied. *PARP9* interacts with *DTX3L*, a liagase that mediates ubiquitination of histone H4 in response to DNA damage, to perform DNA-damage repair [36]. *PARP9* may also promote the migration of B-cells [37]. *PARP14* belongs to a family of enzymes that perform DNA damage-dependent post-translational modification of histone and nuclear proteins which promotes the survival of injured proliferating cells. Of note, *PARP14* is a key regulator of B-cell survival and is highly expressed in multiple myeloma plasma cells. Inhibition of *PARP14* sensitizes cells to anti-myeloma treatments [38], and thus *PARP14* may represent a novel treatment target for other diseases such as SLE. The impact of other hypomethylated sites on autoantibody production still needs to be determined—the biologic function of *RABGAP1L*, for which hypomethylation of CpG sites was also associated with the four autoantibodies under study, has not been well defined. It is suggested to be a tyrosine kinsase that is heavily expressed in myeloid precursors [39]. Further studies are needed to elucidate its role in SLE and SLE-related autoantibody production.

Given the cross-sectional nature of our study, we cannot address causation—whether DNA methylation changes occur before and are relevant to autoantibody production or whether autoantibody production resulting from other disease mechanisms produces DNA methylation changes. However, the identification of new candidate pathways through comprehensive DNA methylation studies provides insight regarding pathogenesis and maintenance of disease states, even if these are not the initial pathways that are perturbed. In addition, since autoantibody titers fluctuate, we considered an individual as autoantibody positive if he/she ever had positive test for a given autoantibody. Thus, the associated methylation changes we identified reflect the individual’s ability to produce that autoantibody and not the individual’s autoantibody titer at a specific time.

All eleven genes/genomic regions identified in this study have been recently found to be hypomethylated in SLE cases compared to healthy controls [11, 12]. All CpG sites except cg10549986 (*RSAD2*) had either moderately or highly statistically significant differences in methylation when comparing SLE cases to healthy controls for three cell populations: CD4+T-cells, CD19+ B-cells, and CD14+ monocytes [12]. Unfortunately, the DNA methylation levels from these studies cannot be directly compared to the results of the current study due to differences in source material and normalization methods. However, our results suggest that the DNA methylation differences seen in the SLE patients when compared to healthy controls may be driven by the subset of autoantibody-producing SLE cases under study, or that further hypomethylation of these genes is associated with more severe disease phenotypes.

The strengths of our study include its large sample size and the careful selection of study subjects which minimizes confounding from differences in gender, ethnicity, and smoking status. Adjusting for disease duration mitigates the effect of the disease itself on DNA methylation status, and estimation and adjustment for leukocyte cell proportions addresses potential differences of the cell composition of the DNA source. Lastly, we have assessed differential DNA methylation broadly—not just at the promoter regions, but throughout genes as well as CpG islands and shores.

While a limitation of our study includes the inability to adjust for medication dose (data not available), we ascertained medication exposure by medical record review and patient questionnaire. Reported (either past or current) use of prednisone, hydroxychloroquine, azathioprine, mycophenolate mofetil, and cyclophosphamide did not differ significantly between the positive and negative patient subgroups for each autoantibody. This is particularly important for methotrexate (p>0.4 for all autoantibody subgroups), since methotrexate use is hypothesized to alter DNA methylation patterns [40, 41]. We were also unable to assess the association between DNA methylation status and disease activity; however, previous studies have suggested that the hypomethylation observed in interferon-related genes in SLE is not related to disease activity [11, 12]. Our study used DNA from peripheral blood cells since it is easily collected in a clinical setting and readily available from our study collection. Therefore, we could not assess whether the differential DNA methylation observed occurs in all leukocytes or occurs in a particular cell population. Studies by other investigators have shown that differential DNA methylation within interferon-related genes is observed in CD4+ T-cells [11] and other cell types [12]. Lastly, we did not compare SLE cases to a healthy control group, so the methylation changes we observed are more informative for autoantibody production among SLE cases rather than SLE disease risk.

In summary, we have identified differentially methylated regions associated with SLE-related autoantibody production, and have shown that these associations are unlikely to be due genetic variation within these regions. Thus, epigenetic studies can provide insight into the mechanisms associated with the clinical heterogeneity of autoimmune disease, and can complement genetic studies to identify biologic pathways that contribute to disease pathogenesis.

## Supporting Information

S1 Fig Volcano plot indicating the DNA methylation difference and associated p-value for each site using the combined dataset (n = 326). (DOCX)

S2 Fig Density of CpG sites on the Illumina HumanMethylation450 BeadChip across the genome. Height of the blue bars indicates the number of CpG sites in that genomic region.
(PNG)

S1 Table DNA methylation sites associated with anti-dsDNA autoantibody production (p<1.07E-07) assessed by Wilcoxon rank sum tests for the combined dataset (n = 326). (XLSX)

S2 Table Assessment of genetic-epigenetic interactions using single nucleotide polymorphism (SNP) association results from a genome-wide association study of anti-dsDNA autoantibody production in SLE. (DOCX)

S3 Table Association results assessed by multivariable linear regression for all SLE-related autoantibodies for all CpG sites associated with at least 1 autoantibody (p<1.07E-07). (XLSX)
