# Supplementary material for: Genome-Wide Assessment of Differential DNA Methylation Associated with Autoantibody Production in Systemic Lupus Erythematosus
Source: PLoS One. 2015 Jul 20;10(7):e0129813. doi: 10.1371/journal.pone.0129813 (PMC4508022; doi:10.1371/journal.pone.0129813)
Supplement: S1 Fig — (DOCX) [file pone.0129813.s001.docx]

**Supplemental Figure 1**. Volcano plot indicating the methylation difference and associated p-value for each site using the combined dataset (n=326).

-log10(p-value)


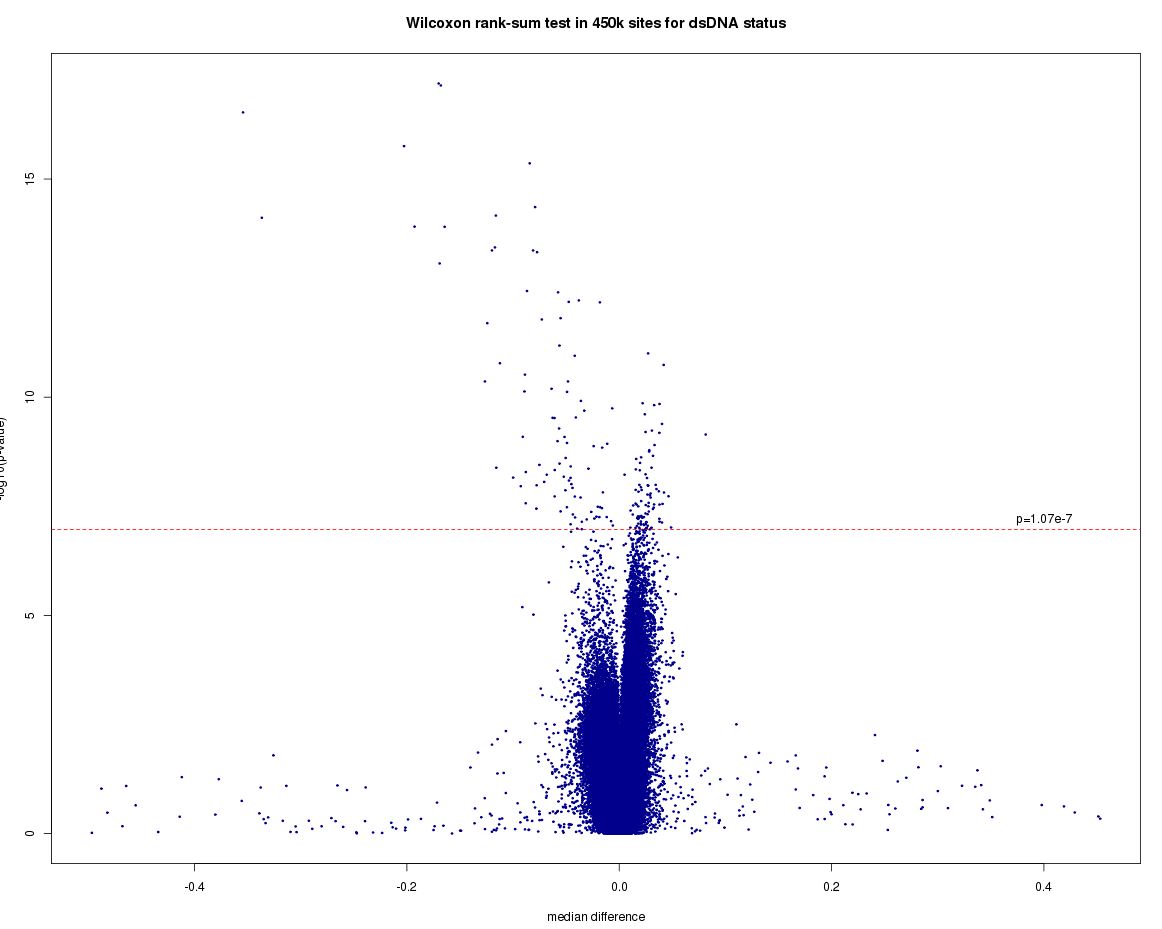


Median difference
