# Supplementary figures and images for: Genome-Wide Assessment of Differential DNA Methylation Associated with Autoantibody Production in Systemic Lupus Erythematosus
Source: PLoS One. 2015 Jul 20;10(7):e0129813. doi: 10.1371/journal.pone.0129813 (PMC4508022; doi:10.1371/journal.pone.0129813)

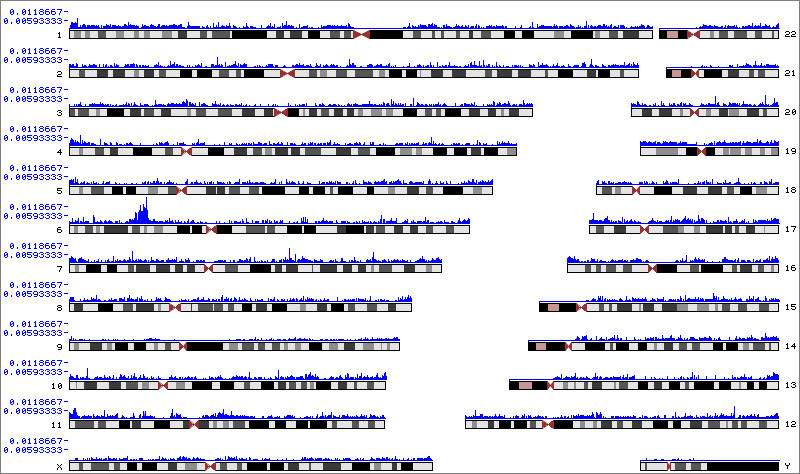

Supplement: S2 Fig — Height of the blue bars indicates the number of CpG sites in that genomic region. (PNG) [file pone.0129813.s002.png]
