# Supplementary material for: Genome-Wide Assessment of Differential DNA Methylation Associated with Autoantibody Production in Systemic Lupus Erythematosus
Source: PLoS One. 2015 Jul 20;10(7):e0129813. doi: 10.1371/journal.pone.0129813 (PMC4508022; doi:10.1371/journal.pone.0129813)
Supplement: S2 Table — (DOCX) [file pone.0129813.s004.docx]

**Supplemental Table 2**. Assessment of genetic-epigenetic interactions using single nucleotide polymorphism (SNP) association results from a genome-wide association study of anti-dsDNA autoantibody production in SLE.

| sites | **chr** | **Gene(s)** | **# SNPs**  **within range^1^** | **# SNPs with p<0.05**  **in anti-anti-dsDNA GWAS** | **SNPs with p<0.01**  **in anti-dsDNA GWAS** |
| --- | --- | --- | --- | --- | --- |
| cg06872964, cg05696877 | 1 | *IFI44L* | 97 | 7 |  |
| cg13130398, cg07285983 | 1 | *RABGAP1L* | 66 | 2 |  |
| cg10959651, cg10549986 | 2 | *RSAD2* | 118 | 3 |  |
| cg17326313 | 2 | *EIF2AK2* | 73 | 0 |  |
| cg08122652, cg00959259, cg01948202 | 3 | *PARP9, DTX3L, PARP14* | 50 | 0 |  |
| cg06981309 | 3 | *PLSCR1* | 64 | 2 |  |
| cg05552874 | 10 | *IFIT1* | 85 | 1 |  |
| cg19789466 | 12 | *OAS1* | 104 | 3 | rs1650060 (p=0.0095) |
| cg07839457, cg16411857 | 16 | *NLRC5* | 80 | 6 | rs729334 (p=0.0046) |
| cg21549285 | 21 | *MX1* | 161 | 11 |  |

^1^ Number of SNPs located 250 kb up- and down-stream of the differentially methylated sites.
